# The Use of Repertories

**Published:** 1892-02

**Authors:** 


THE USE OF REPERTORIES.—This paper, by Dr. Holmes, on page 36, was originally intended for this journal, but was published in the *Medical Advance* as part of the proceedings of the I. H. A. It has since been rewritten and enlarged for our pages.

